# Epitranscriptome changes triggered by ammonium nutrition regulate the proteome response of maritime pine roots

**DOI:** 10.3389/fpls.2022.1102044

**Published:** 2022-12-22

**Authors:** Francisco Ortigosa, César Lobato-Fernández, Juan Antonio Pérez-Claros, Francisco R. Cantón, Concepción Ávila, Francisco M. Cánovas, Rafael A. Cañas

**Affiliations:** ^1^ Grupo de Biología Molecular y Biotecnología de Plantas, Departamento de Biología Molecular y Bioquímica, Universidad de Málaga, Málaga, Spain; ^2^ Departamento de Ecología y Geología, Universidad de Málaga, Málaga, Spain; ^3^ Integrative Molecular Biology Lab, Universidad de Málaga, Málaga, Spain

**Keywords:** nitrogen nutrition, ammonium, epitranscriptomics, ONT sequencing, *Pinus pinaster*, translation

## Abstract

Epitranscriptome constitutes a gene expression checkpoint in all living organisms. Nitrogen is an essential element for plant growth and development that influences gene expression at different levels such as epigenome, transcriptome, proteome, and metabolome. Therefore, our hypothesis is that changes in the epitranscriptome may regulate nitrogen metabolism. In this study, epitranscriptomic modifications caused by ammonium nutrition were monitored in maritime pine roots using Oxford Nanopore Technology. Transcriptomic responses mainly affected transcripts involved in nitrogen and carbon metabolism, defense, hormone synthesis/signaling, and translation. Global detection of epitranscriptomic marks was performed to evaluate this posttranscriptional mechanism in un/treated seedlings. Increased N^6^-methyladenosine (m^6^A) deposition in the 3’-UTR was observed in response to ammonium, which seems to be correlated with poly(A) lengths and changes in the relative abundance of the corresponding proteins. The results showed that m^6^A deposition and its dynamics seem to be important regulators of translation under ammonium nutrition. These findings suggest that protein translation is finely regulated through epitranscriptomic marks likely by changes in mRNA poly(A) length, transcript abundance and ribosome protein composition. An integration of multiomics data suggests that the epitranscriptome modulates responses to nutritional, developmental and environmental changes through buffering, filtering, and focusing the final products of gene expression.

## Introduction

Discoveries in the nascent field of molecular biology culminated in the central dogma of molecular biology during the 1970s (Crick, 1970). Currently, it is known that transcription and translation processes are not always directly linked. Multiple factors intervene in the gene response and its regulation. Two good examples of this are long noncoding RNAs (lncRNAs) (Liu et al., 2015) and microRNAs (miRNAs) (Paul et al., 2015). These types of RNA regulate important aspects of both development and response to external stimuli in plants (Liu et al., 2015; Paul et al., 2015). However, these studies represent only some aspects of overall RNA metabolism. In recent years, an increasing interest has focused on determining the biological role of RNA chemical modifications emerging as a new field of study under the term epitranscriptomics. These modifications are found in all RNA types, such as transfer RNAs (tRNAs), ribosomal RNAs (rRNAs), messenger RNAs (mRNAs) and small RNAs (RNAs) (Xiong et al., 2017). To date, more than 160 different modifications have been identified in RNA (Shen et al., 2019). In *Arabidopsis thaliana*, m^7^G, m^6^A, m^1^A, m^5^C, hm^5^C, and uridylation have been identified as modifications in mRNA (Shen et al., 2019). The marriage between classical detection techniques and high-throughput sequencing has allowed to determine N^6^-methyladenosine (m^6^A) as the most prevalent chemical modification in mRNAs, both in animals and plants (Fray and Simpson, 2015). Transcriptome-wide analysis revealed that the m^6^A mark in transcripts is predominantly located near the stop codon and throughout the 3′ untranslated region (UTR) (Shen et al., 2019). An m^6^A methylation motif (RR**A**CH [R = A/G; H = A/U/C; **A** = m^6^A]) that is conserved between plants and other eukaryotic organisms has been described (Shen et al., 2019). m^6^A deposition, recognition and elimination are carried out by different proteins commonly called writers, readers, and erasers, respectively (Shen et al., 2019). Several cellular functions have been observed to be affected by m^6^A modification, such as mRNA stability (Wei et al., 2018) or translational efficiency (Luo et al., 2014). In addition, proper m^6^A deposition has been reported to be essential during *Arabidopsis* embryo development (Zhong et al., 2008) and to take part in biotic and abiotic plant stress responses (Martínez-Pérez et al., 2017; Anderson et al., 2018), fruit ripening (Zhou et al., 2019), flowering transition (Duan et al., 2017), leaf morphogenesis (Arribas-Hernández et al., 2018), trichome development (Bodi et al., 2012) and apical shoot meristem development (Shen et al., 2016).

Nitrogen (N) is an essential element for plant growth and development, and a key component of cellular constituents such as nucleic acids, proteins, and chlorophylls (Hawkesford et al., 2012). However, little is known about the potential role of the epitranscriptome in the regulation of N nutrition, with only a recent study on the involvement of m^6^A in nitrate assimilation and signaling in *Arabidopsis* (Hou et al., 2021). In soils, plants can take up N inorganic forms such as nitrate ( ${\text{NO}}_{3}^{-}$ ) or ammonium ( ${\text{NH}}_{4}^{+}$ ) and organic forms such as urea, peptides, and amino acids (Jia and von Wirén, 2020). Plants such as rice, tea and maritime pine prefer ${\text{NH}}_{4}^{+}$ over ${\text{NO}}_{3}^{-}$ as the main N source (Sasakawa and Yamamoto, 1978; Ruan et al., 2016; Ortigosa et al., 2020). In many plants, important changes in the transcriptome, proteome and metabolome have been described in relation to nitrogen nutrition and have mainly focused on the supply of ${\text{NO}}_{3}^{-}$ and ${\text{NH}}_{4}^{+}$ (Patterson et al., 2010; Yang et al., 2018; Ravazzolo et al., 2020). In this way, it has been observed that these N forms trigger both shared and differential responses involving different pathways and many result in phenotypic differences such as specific changes in the root system architecture (RSA) and growth (Jia and von Wirén, 2020). Therefore, it is reasonable to hypothesize that some of these described responses to N nutrition may be influenced by epitranscriptome regulatory processes.

Oxford Nanopore Technology (ONT) is a third-generation sequencing platform that is currently the only option for direct sequencing of RNA samples without the requirement of reverse transcription and amplification steps (Parker et al., 2020). These features are of great relevance in transcriptomics as they reduce sequencing biases and maintain nucleoside modifications that enable epitranscriptomic studies (Gao et al., 2021).

Research efforts of this work were focused on maritime pine (*Pinus pinaster* Aiton), which is a conifer typically found in the western Mediterranean region. In these areas maritime pine constitutes extensive forests being mainly located in Portugal, Spain, and France where it has been used for raw material obtention such as timber, pulp, and resin. This tree is a model species for research on functional genomics, drought resistance or nitrogen nutrition and metabolism in conifers (Sterck et al., 2022; Ávila et al., 2022). Maritime pine is a plant with a preference for ammonium over nitrate nutrition, which promotes an increase in biomass accumulation, especially in the roots where a higher number of lateral roots has been observed (Ortigosa et al., 2020; Ortigosa et al., 2022). In addition, ammonium supply promotes transcriptomic changes in several phytohormone-related transcripts such as *ACC oxidase*, as well as localization of phytohormones in root tips (Ortigosa et al., 2022). One of its main attractions is that it can provide an evolutionary insight into different processes studied in other model organisms, since the maritime pine is included in the group of gymnosperms whose appearance on Earth is estimated to be about 100 million years before the appearance of angiosperms (Clarke et al., 2011).

The aim of the present work is to shed light on the short-term response of maritime pine roots to ${\text{NH}}_{4}^{+}$ nutrition, elucidating what kind of regulatory relationship exists between transcriptomics, epitranscriptomics and proteomics. For this purpose, cutting edge and commonly used omics approaches, such as comprehensive transcriptome analyses by direct RNA sequencing (DRS) using ONT, epitranscriptomic modification detection focused on m^6^A assisted by the ONT platform, and quantitative proteomic, were combined in the present study.

## Material and methods

### Plant material

Seeds from maritime pine (*Pinus pinaster* Aiton) from “Sierra Segura y Alcaraz” (Albacete, Spain) were provided by the *Área de Recursos Genéticos Forestales* of the Spanish *Ministerio de Agricultura, Pesca y Alimentación*. Maritime pine seed germination was carried out following the protocol described in (Cañas et al., 2006). Seedlings were grown in vermiculite in plant growth chambers under 16 h light photoperiod, a light intensity of 100 μmol m^−2^ s^−1^, constant temperature of 25 °C and watered twice a week with distilled water. One-month old maritime pine seedlings were used for the experiment. Pine seedlings were randomly subdivided into two different groups, relocated into forestall seedbeds and watered with 80 mL distilled water. After three days of acclimation, the control group was irrigated with 80 mL of water (C) and the experimental group with 80 mL of 3 mM NH_4_Cl. This ammonium concentration (3mM) is N-sufficient for the growth of maritime pine (Canales et al., 2010). Root samples were collected at 24 hours post-irrigation and immediately frozen in liquid N. This experiment was carried out three independent times. The adequate development of each experiment was verified through the gene expression analysis by RT-qPCR of two control genes, *PpAMT1.3* and *PpAMP1* following previous results (Canales et al., 2011; Castro-Rodríguez et al., 2016). The sequences of this genes can be found in Genbank under the following accession numbers: KC807909 (PpAMT1.3) and HM210085 (PpAMP1).

### Metabolite profile

The metabolites for ^1^H-NMR analysis were extracted following the protocol previously described by Kruger et al. (2008) and according to Ortigosa et al. (2020). Extended method descriptions are in the **Supplementary Methods**. All data and results have been included in **Supplementary Figure 1** and **Supplementary Dataset 1**.

### Total RNA isolation

Total root RNA from maritime pine seedlings was isolated following the protocol described by Liao et al. (2004) and modified by Canales et al. (2012). The RNA concentration and purity were determined *via* spectrophotometry on a Nanodrop ND-1000 (Thermo Scientific, Waltham, MA, USA). Purity was determined through the 260/280 and 260/230 ratios. RNA quality was also determined in a Bioanalyzer 2100 (Agilent, Santa Clara, CA, USA). The concentration was verified with a Qubit 4 Fluorometer (Invitrogen, Paisley, UK) and Qubit RNA BR, Broad-Range, Assay Kit (Cat. No. Q10210, Invitrogen, Paisley, UK).

### mRNA isolation and preparation

Samples with a RIN value > 7 were selected to mRNA isolation. The poly(A)-RNA isolation was performed using Dynabeads™ mRNA Purification Kit (Cat. No. 61006, Invitrogen, Paisley, UK) following the manufacturer’s instructions. This process was carried out twice per sample to avoid rRNA contamination. poly(A)-RNA quality was determined in a Bioanalyzer 2100 (Agilent, Santa Clara, CA, USA). The concentration was verified with a Qubit 4 Fluorometer (Invitrogen, Paisley, UK) and Qubit RNA HS, High Sensitivity, Assay Kit (Cat. No. Q32852, Invitrogen, Paisley, UK).

### Direct RNA sequencing (DRS) and differential epitranscriptomic analysis

Nanopore libraries for DRS were prepared from 1.65 up to 2.18 µg of isolated poly(A)-RNA using the Nanopore Direct RNA Sequencing kit (Cat. No. SQK-RNA001, Oxford Nanopore Technologies, ONT, Oxford, UK) according to manufacturer’s instructions. The DRS libraries were loaded onto a R9.4 SpotON Flow Cells (Cat. No. FLO-MIN106D, Oxford Nanopore Technologies, Oxford, UK) and sequenced until complete depletion of the nanopores. Extended method descriptions are in the **Supplementary Methods**. The DRS data have been deposited in the NCBI's Gene Expression Omnibus (Edgar et al., 2002) and are accessible through GEO Series with the accession number GSE174830 (https://www.ncbi.nlm.nih.gov/geo/query/acc.cgi?acc=GSE174830). Basecalling was carried out with ONT Guppy software (https://community.nanoporetech.com). The resultant reads were filtered by quality (Q>9). Read alignment was made with minimap2 software (Li, 2018) using root transcriptome of maritime pine as reference (Ortigosa et al., 2022). The alignment parameters were adjusted for DRS (-*uf* and -*k14*). Differentially expressed (DE) transcripts were identified using the edgeR package for R, the transcripts were normalised by count per million mapped reads (cpm) and filtered (2 cpm in at least 2 samples) (Robinson et al., 2010). The transcripts with False Discovery Rate < 0.05 (FDR < 0.05) were considered as differentially expressed.

ONT-DRS reads were used for *de novo* modification detection with the TOMBO software (Stoiber et al., 2017). The total mapped reads per base and the number of modified bases in each position were obtained using the *text_output_browser_file* method with the options *coverage* and *fraction*. Only the positions with at least 50 mapped reads were employed for subsequent analyses. A Fischer exact test was carried out for each transcript position to determine the differential expression of modified bases among control and ${\text{NH}}_{4}^{+}$ supplied samples. The transcript positions with a *P-*value < 0.05 and an absolute logFC > 0.5 were considered as differentially modified.

*Transdecoder* software (https://github.com/TransDecoder/transdecoder.github.io) were used to determine modification positions and nucleobases in the transcripts of the reference transcriptome. Identification of m^6^A sites were carried out with the bioinformatic pipeline *Nanom6A* using default parameters (Gao et al., 2021). The length of poly(A) tails was determined using *Nanopolish* 0.11.1 software package (https://github.com/adbailey4/nanopolish) with the *polya* function.

### Functional annotation and enrichment analyses

The transcriptome was functionally annotated with BLAST2GO (Götz et al., 2008) using DIAMOND software with *blastx* option (Buchfink et al., 2015) against the NCBI’s plants-*nr* database (NCBI Resource Coordinators, 2016). Blast results were considered valid with e-value < 1.0E-6. Singular enrichment analysis (SEA) of the GO terms was made in the AGRIGO v2.0 web tool under standard parameters using as GO term reference the whole assembled transcriptome annotation (Tian et al., 2017). Representative enriched GO was determined using REVIGO (Supek et al., 2011).

### RT-qPCR

The cDNA synthesis was performed using 1 μg of total RNA and iScript™ cDNA Synthesis Kit (Bio-Rad, Hercules, CA, USA) following manufacturer’s instructions. The qPCR primers were designed following the MIQE guidelines (Bustin et al., 2009). The primers are listed in the **Supplementary Table 1**. qPCRs were carried out using 10 ng of cDNA and 0.4 mM of primers and 2X SsoFast™ EvaGreen^®^ Supermix (Cat. No. 1725204, Bio-Rad, Hercules, CA, USA) in a total volume of 10 μL. Relative quantification of gene expression was performed using thermocycler CFX 384™ Real-Time System, Bio-Rad, Hercules, CA, USA). The qPCR program was: 3 min at 95°C (1 cycle), 1 s at 95°C and 5 s at 60°C (50 cycles) and a melting curve from 60 to 95°C, to generate the dissociation curve in order to confirm the specific amplification of each individual reaction. The analyses were carried out as described by Cañas et al. (2014) using the MAK3 model in the R package *qpcR* (Ritz and Spiess, 2008). Expression data were normalized to two reference genes, SKP1/ASK1 and SLAP that were previously tested for RT-qPCR experiments in maritime pine (Granados et al., 2016). The qPCR analyses were made with three biological replicates and three technical replicates per sample.

### Validation of differential deposition of m^6^A by RT-qPCR

The validation of the differential deposition of m^6^A in the transcripts were made using the SELECT method (Xiao et al., 2018). A differential cDNA synthesis was made per each transcript with 30 ng of total RNA. qPCR determinations were made with 2 μL of cDNA. The expression level of each transcript was determined in parallel by RT-qPCR and their result were used to normalize the SELECT results. The primers are listed in the **Supplementary Table 1**. Extended method description can be found in the **Supplementary Methods**.

### Differential proteomics analysis

The proteins were extracted following the protocol described by González Fernández et al. (2014). The extractions were carried out with 200 mg of sample. Protein content was determined using a commercially kit (Cat. No. 5000006, Protein Assay Dye Reagent; Bio-Rad, CA, USA) and bovine serum albumin as a standard (Bradford, 1976). Protein extracts were cleaned-up in 1D SDS-PAGE at 10% polyacrilamyde as described in Valledor and Weckwerth, 2014. Protein bands were cut off, diced, and kept in water at 4°C until digestion.

Protein digestion and nLC-MS2 analysis were carried out in the Proteomics Facility at Research Support Central Service, University of Cordoba. Nano-LC was performed in a Dionex Ultimate 3000 nano UPLC (Thermo Scientific, Waltham, MA, USA) with a C18 75 μm x 50 Acclaim Pepmam column (Thermo Scientific, Waltham, MA, USA). Eluting peptide cations were converted to gas-phase ions by nano electrospray ionization and analyzed on a Thermo Orbitrap Fusion (Q-OT-qIT, Thermo Scientific) mass spectrometer operated in positive mode. Extended method descriptions are in the **Supplementary Methods**.

Root transcriptome from *Pinus pinaster* was translated into the six open reading frames with *transeq* tool (Madeira et al., 2019). The output peptides chains were filtered by length, deleting those less than 50 amino acids (Romero-Rodríguez et al., 2014). To reduce the redundancy of proteins in the database, CD-HIT-EST with a 99% identity filter was used (Fu et al., 2012). The raw data were processed using Proteome Discoverer (version 2.3, Thermo Scientific). MS2 spectra were searched with SEQUEST engine against the reference proteome database. *In silico* peptide lists were created using the followings settings: trypsin digestion, a maximum of two missed internal cleavage sites per peptide, precursor mass tolerance of 10 ppm and fragment mass tolerance of 0.75 Da per fragment ions. Only peptides with a high confidence (FDR ≤ 0.01) and minimum XCorr of 2 were selected. The identified proteins were filtered by a minimum of two different peptides. *Minora* algorithm was used to determine relative quantification. The protein identification with redundancy is considered by Proteome Discoverer and SEQUEST software. Proteins sharing peptides were grouped and all those groups without a unique peptide were removed. Only proteins detected in 5 of the 6 samples were considered for subsequent analyses. The resultant proteins were annotated using BLAST with *blastp* option against NCBI’s non-redundant database (Camacho et al., 2008) and BLAST2GO software (Götz et al., 2008). Proteins with the same sequence annotation and quantification profile across the samples was considered the same protein selecting the protein with the longest sequence. Normalized data from Proteome Discoverer software were used for a differential expression analysis using the edgeR package for R (Robinson et al., 2010). Only the proteins with *P*-value < 0.05 were considered as differentially expressed. The mass spectrometry proteomics data have been deposited to the ProteomeXchange Consortium via the PRIDE partner repository (Vizcaino et al., 2013) with the Data identifier PXD025331 and 10.6019/PXD025331.

## Results

### Direct RNA sequencing (DRS)

**Figure 1:**
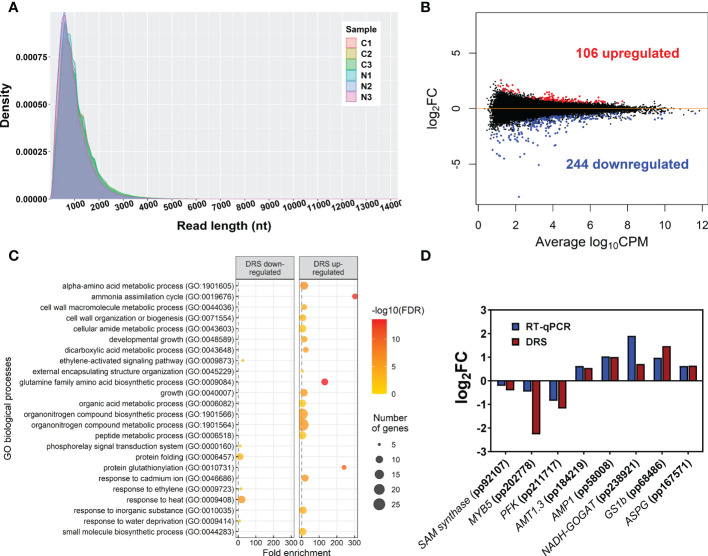
Main DRS transcriptomics results. **(A)** Size distribution of reads obtained through DRS-ONT sequencing. **(B)** MA plot containing all detected transcripts. Blue points correspond to significantly downregulated transcripts in maritime pine roots at 24 h after the treatment with 3 mM ammonium. Red points correspond to significantly upregulated transcripts in maritime pine roots at 24 h after the treatment with 3 mM ammonium. **(C)** Significant GO terms from significant DE transcripts after a SEA analysis. **(D)** Comparison between DRS and RT-qPCR results from different transcripts with significant differential expression in the DRS analysis.

The global sequencing results are shown in **Table 1**. The mean read sizes were between 908 bp to 1059 bp (**Figure 1A**). The longest reads ranged from 10298 bp to 14299 bp. Differential expression analyses identified 350 differentially expressed (DE) transcripts. From the total DE transcripts obtained, 106 were upregulated and 244 were downregulated (**Figure 1B**; **Supplementary Dataset 2**). Singular enrichment analysis (SEA) was performed individually for each gene expression regulation (up- and downregulated) to classify the biological functions under ${\text{NH}}_{4}^{+}$ nutrition. The SEA global results are shown in **Supplementary Dataset 3**. Upregulated transcripts were significantly enriched with GO terms for Biological Process (BP) such as *ammonia assimilation cycle*, *protein glutathionylation*, and *developmental growth*; for Cellular Component (CC) such as *chloroplast stroma* and *cytosolic ribosome*; and for Molecular Function (MF) such as *glutamate synthase (NADH) activity*, and *phospholipase activity* (**Figure 1C**; **Supplementary Figure 2**). The downregulated transcripts were enriched in BP terms such as *protein folding* and *ethylene-activated signaling pathway* and MF terms such as *transcription coactivator activity*, *mRNA binding*, and *chaperone binding*. A more detailed study of the upregulated transcriptomic response revealed that *PpGS1b* (pp_68481) and *PpNADH-GOGAT* (pp_238920) were upregulated. Interestingly, transcripts for genes involved in defense-related response were upregulated: *PpAMP1* (pp_58005, pp_58008), different class IV chitinases (pp_239593, pp_239598, pp_239600, pp_117809), different splicing coding forms of patatin-like protein 2 (pp_71017, pp_71018, pp_71019, pp_71020, pp_71022), a PR-1 pathogenesis-related protein (pp_87427), a defensin coding transcript (pp_92119) and an RPW8 domain-containing protein (pp_142311) (**Supplementary Dataset 2**). Cell wall-related transcripts were also upregulated, such as those encoding expansin-A18 (pp_134987), nonclassical arabinogalactan protein 30 (pp_66323), probable prolyl 4-hydroxylase 4 (pp_235715), xyloglucan:xyloglucosyl transferase (pp_68519) and different forms of xyloglucan endotransglucosylase/hydrolase (pp_66707, pp_66708, pp_68517), was also observed. In contrast, the downregulation of different transcription factors (TFs) was observed (**Supplementary Dataset 2**), such as ethylene response factors (ERFs) (pp_58625, pp_58626, pp_86737, pp_96228, pp_96234), a trihelix transcription factor (pp_59947), and a MYB coding transcript (pp_202778). The repression of different splicing forms of an auxin-repressed protein/dormancy-auxin associated protein coding transcript (pp_58457, pp_58458, pp_58459, pp_58461, pp_58462, pp_58463), the SnRK1 regulator FCS-like zinc finger transcript (pp_202096), and transcripts encoding carbohydrate metabolism enzymes such as pyruvate decarboxylase (pp_78343, pp_123611, pp_126347) and sucrose synthase (pp_144843) was also observed. The differential expressions observed in DRS was confirmed by RT-qPCR analyses of seven transcripts including *SAM synthase, MYB5, PFK, AMP1, NADH-GOGAT, GS1b*, and *ASPG*. The results showed the same trend between the DRS and RT-qPCR results for all DE transcripts, with some differences in the logFC values (**Figure 1D**).

**Table 1:** Direct RNA sequencing information for each sample run.

Sample	Length read (mean, bp)	Length read (median, bp)	Maximum length read (bp)	Maximum quality per sample
C1	1022.96	887	14299	27.94
C2	1009.87	854	11465	29.37
C3	1059.41	889	10808	30.20
N1	1031.77	886	12834	14.32
N2	907.56	768	10951	29.96
N3	927.18	786	10298	30.50
**Mean**	993.13	845	11775.83	27.05

### Differential DRS epitranscriptomics

**Figure 2:**
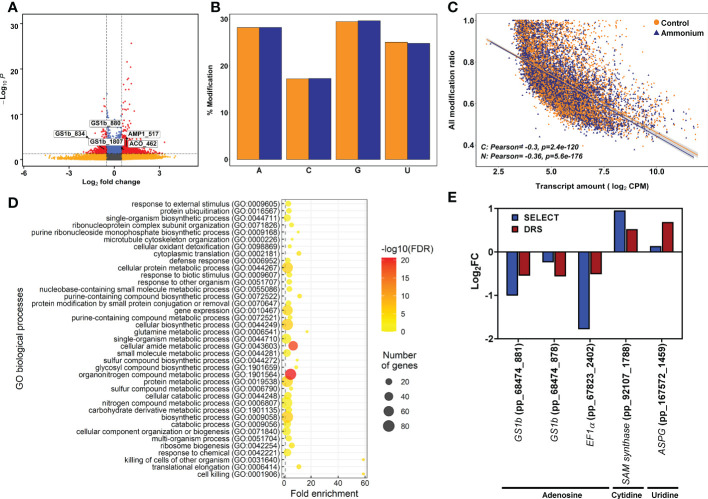
Epitranscriptomics modification results from Tombo software analysis. **(A)** Volcano plot of the detected epitranscriptomics modifications. Yellow points correspond to modifications with *P*-values lower than 0.05 but with an absolute logFC value lower than 0.5. Blue points correspond to modifications with an absolute logFC value higher than 0.5 but with *P*-values higher than 0.05. Red points correspond to significant DE modifications with an absolute logFC value higher than 0.5 and *P*-values lower than 0.05. **(B)** Percentage of detected modifications per nucleobase. Yellow columns correspond to control samples. Purple columns correspond to 3 mM ammonium treated samples. **(C)** Scatter plot and correlation between detected modifications using Tombo software and transcript amounts determined by DRS sequencing. **(D)** Significant GO terms from significant DE modifications after a SEA analysis. **(E)** Comparison between DRS and SELECT results from different transcript modified positions with significant differential modification in the Tombo analysis.

To determine the differential epitranscriptomic marks, DRS results were explored to identify the chemically modified nucleosides in the mRNAs using Tombo software. The number of putative modified nucleosides in the transcripts ranged from 722,655 in sample C1 to 4,063,005 in sample N3 (**Supplementary Datasets 4–9**). After differential deposition analysis, 513 significant differentially modified nucleosides were obtained in 283 transcripts (**Figure 2A**; **Supplementary Dataset 10**). There were 221 undermodified positions in 161 transcripts and 292 overmodified positions in 184 transcripts. Among them, 58 transcripts had significant over- and undermodified positions, including *PpGS1b* (pp_68474) and *translationally-controlled tumor protein* (*TCTP*, pp_72505) (**Supplementary Dataset 11**). The percentage of modifications for each kind of nucleoside was similar for adenosine, guanosine, and uridine (28%, 29% and 25%) but lower for cytidine (17%), without any obvious difference between sample conditions (**Figure 2B**). When the global set of modification ratios and transcript amounts were compared (**Figure 2C**), significant and negative correlations were found to be stronger under ${\text{NH}}_{4}^{+}$ supply (-0.36) than under the control (-0.30) (**Figure 2C**). Modification ratios of individual nucleosides compared to transcript amounts were also similar (**Supplementary Figure 3**).

The functions of the transcripts with DE modifications were analyzed using SEA (**Figure 2D**; **Supplementary Figure 4** and **Supplementary Dataset 12**). A total of 116 significant GO terms were obtained. At the BP level, the main functions were related to the terms *ribosome biogenesis*, *translation*, *protein ubiquitination*, and *glutamine metabolic process*. Similarly, the *ribosome* term was the main function at the CC level. Finally, the terms *translation factor activity*, *ubiquitin protein ligase binding*, and *endopeptidase inhibitor activity* were the main functions at the MF level.

The differential modifications were verified using SELECT. This qPCR-based technique was initially designed to determine differential deposition of m^6^A in total RNA mixtures. However, nucleoside modifications putatively detected in cytidine and uridine by Tombo were also detected with SELECT (**Figure 2E**). The obtained results for each position showed a similar trend between the differential epitranscriptomic results from SELECT and Tombo (**Figure 2E**).

### m^6^A identification

**Figure 3:**
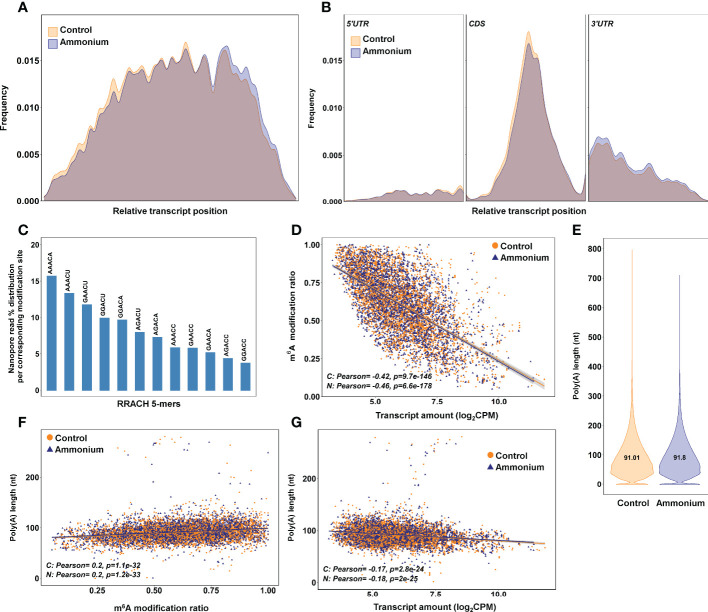
m^6^A identification results using the bioinformatics pipeline Nanom6A. **(A)** Distribution of the identified m^6^A all along the transcripts. **(B)** Distribution of the identified m^6^A along each part of the transcripts: 5’UTR, CDS and 3’UTR respectively. **(C)** Percentage of identified m^6^A for each different variant (kmer) of the consensus sequence RRACH for adenine methylation. **(D)** Scatter plot and correlations between m^6^A modification ratio and transcript amount from DRS sequencing. **(E)** Poly(A) length of the transcripts identified in the DRS sequencing. **(F)** Scatter plot and correlations between poly(A) length and m^6^A modification ratio. **(G)** Scatter plot and correlations between poly(A) length and transcript amount from DRS sequencing.

The bioinformatic pipeline Nanom6A was used to specifically identify m^6^A modifications in the RRACH sites from DRS data. Statistical analysis identified 176 nucleosides with significant (*P*-value < 0.05) differential deposition of m^6^A, but only 29 were considered as having a |logFC| > 0.5 (**Supplementary Dataset 13**). The distribution of m^6^A sites along the full-length transcripts showed a higher accumulation of marks over two-thirds of the relative length in control samples while a higher accumulation of marks in the final portion of the transcripts was observed in ${\text{NH}}_{4}^{+}$ -treated samples (**Figure 3A**). A more detailed distribution study showed that m^6^A sites were more abundant in the 5’-UTR and coding (CDS) regions under the control condition, while m^6^A sites tended to be more abundant in the 3’-UTR regions of transcripts isolated from ${\text{NH}}_{4}^{+}$ -treated seedlings (**Figure 3B**). The m^6^A frequency was higher in the CDS, mainly in the central portion, and at the beginning of the 3’-UTR regions, but it was lower at the transcript ends (**Figures 3A, B**).

The most predominant RRACH sequence was AAACA (>15%), while GGACC was the least abundant (< 5%) (**Figure 3C**). The comparison between m^6^A modification ratios and transcript amounts showed significant negative correlations for the control (-0.42) and ${\text{NH}}_{4}^{+}$ conditions (-0.46) (**Figure 3D**). The lengths of the poly(A) tails were determined from DRS data (**Figure 3E**). As expected, most of the poly(A) tails had a size between 40-250 nt with similar means in both conditions, 91.01 nt and 91.8 nt. The poly(A) tail lengths had significant positive correlations with m^6^A ratios in both conditions (0.2) (**Figure 3F**). Significant but lower positive correlations were found when poly(A) tail lengths were compared with global and nucleoside modification ratios obtained with Tombo (**Supplementary Figure 5**). Finally, the poly(A) tail lengths and transcript amounts exhibited significant negative correlations for the control (-0.17) and ${\text{NH}}_{4}^{+}$ conditions (-0.18) (**Figure 3G**).

### Differential proteomics

**Figure 4:**
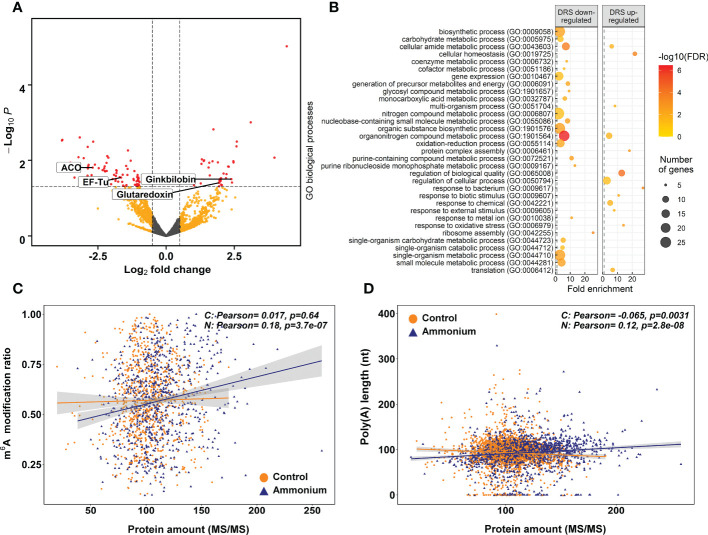
Differential expression proteomics results. **(A)** Volcano plot of the identified proteins. Yellow points correspond to modifications with *P*-values lower than 0.05 but with an absolute logFC value lower than 0.5. Red points correspond to significant DE modifications with an absolute logFC value higher than 0.5 and *P*-values lower than 0.05. **(B)** Significant GO terms from significant DE proteins after a SEA analysis. **(C)** Scatter plot and correlations between m^6^A modification ratios and protein amounts. **(D)** Scatter plot and correlations between transcript amounts from DRS sequencing and protein amounts.

A total of 2,385 proteins were identified in the shotgun proteomics analysis (**Supplementary Dataset 14**). Among the identified proteins, 114 were differentially regulated by ${\text{NH}}_{4}^{+}$ : 38 were more abundant, while 76 were less represented (**Figure 4A**; **Supplementary Dataset 14**). To elucidate the biological roles of the identified proteins, SEA analyses were performed (**Figure 4B**; **Supplementary Figure 6** and **Supplementary Dataset 15**). The upregulated proteins showed as representative BP terms *cell redox homeostasis*, *protein complex assembly*, and *translation*. At CC level, *ribosome*, *nucleolus* and *chloroplast* were the enriched terms. Finally, structural *constituent of ribosome* and *enzyme regulator activity* were the significant MF terms. Among the downregulated proteins, the representative enriched functions were *ribosome assembly* and *translation* among the BP terms. At the CC level, the significant terms most interesting was *ribosome*. *Structural constituent of ribosome* and *GTPase activity* were the enriched MF terms.

The putative relationship between the m^6^A modification ratio and protein abundance was determined through Pearson correlation analysis (**Figure 4C**). In ${\text{NH}}_{4}^{+}$ -treated seedlings, a significant positive correlation was observed (0.18), while there was no correlation in control seedlings. The same effect was observed between nucleoside modification ratios from Tombo and protein amounts (**Supplementary Figure 7**). In addition, similar correlation results were obtained when poly(A) tail lengths and protein amounts were compared, and only ${\text{NH}}_{4}^{+}$ -treated roots had a significant positive correlation (0.12) (**Figure 4D**).

### Integration of data from omics approaches

**Figure 5:**
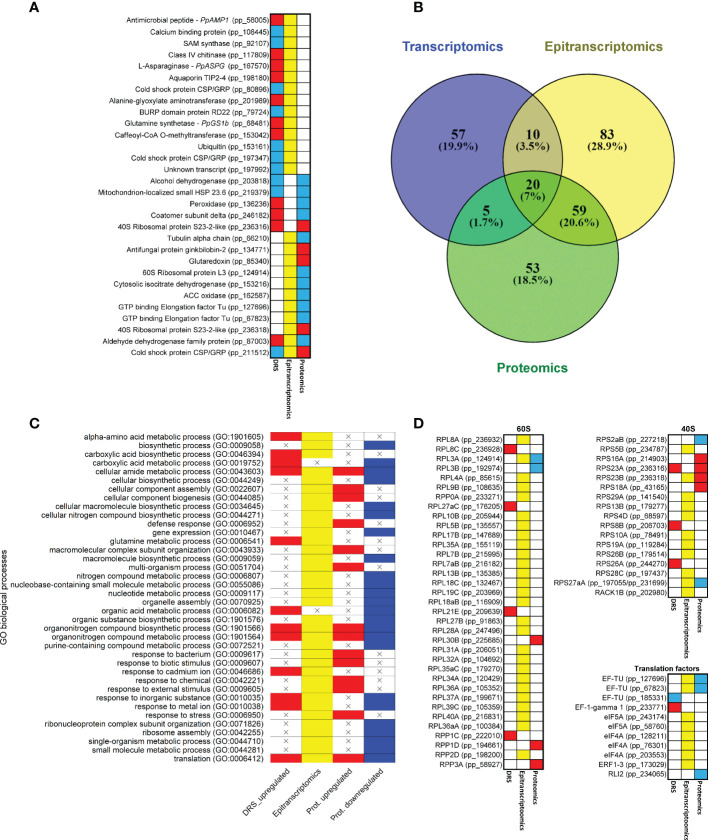
Common results among different omics approaches. **(A)** Genes with significant expressions in more than one omics approach. Red boxes correspond to upregulated transcripts and proteins. Blue boxes correspond to downregulated transcripts and proteins. Yellow boxes correspond to significant differentially modified positions in the transcripts. **(B)** Venn diagram of significant GO terms in the different omics approaches. **(C)** Heatmap of significant GO terms shared by at least two omics approaches. **(D)** Heatmap of genes that produce proteins involved in ribosome and translation. Ribosomal protein equivalences obtained from Martinez-Seidel et al. (2020). Red boxes correspond to upregulated transcripts and proteins. Blue boxes correspond to downregulated transcripts and proteins. Yellow boxes correspond to significant differentially modified positions in the transcripts.

The results of the different omics approaches employed in the present work were integrated to explore possible regulatory steps in response to ${\text{NH}}_{4}^{+}$ supply in maritime pine. The global data showed 30 different elements/genes with significant results based on at least two approaches; 14 of them were significant in DRS and epitranscriptomics, 5 in DRS and proteomics, 9 in epitranscriptomics and proteomics, and 2 in all approaches (**Figure 5A**; **Supplementary Dataset 16**). Altogether, the genes/proteins identified were involved in N metabolism (*PpASPG*, *PpGS1b*, alanine-glyoxylate aminotransferase and isocitrate dehydrogenase), defense (*PpAMP1*, a ginkbilobin and a chitinase), oxidative stress response (alcohol dehydrogenase, aldehyde dehydrogenase, peroxidase and glutaredoxin), translation (ribosomal proteins and elongation factors) and RNA binding (cold shock proteins and a BURP domain protein RD22). Among them, the presence of the 1-aminocyclopropane-1-carboxylate (ACC) oxidase, which had a putative modification at the position 462 on the contig, must be highlighted. This epitranscriptomic mark was overexpressed (logFC 0.84) under ${\text{NH}}_{4}^{+}$ nutrition, while the ACC oxidase protein was underexpressed (logFC -2.69). Similarly, changes in transcript level, protein level and transcript modification ratio can show opposite trends with variations between genes. For the aldehyde dehydrogenase, the transcript expression and modification were overexpressed (0.58 and 0.73, respectively), but protein expression was underexpressed (logFC -1.20). However, for the CSP/GRP (pp_211512 and pp_211516), transcript accumulation was underexpressed (-0.43 and -0.7) and the epitranscriptomic modifications and protein expression were overexpressed (0.88-0.73 and 2.11 respectively).

The comparison between the significant GO terms in the omics approaches revealed that 94 of the 287 GO terms were shared (**Figures 5B, C**; **Supplementary Dataset 17**). Interestingly, 20 (7%) of them were common to the three sets of results. The epitranscriptomics and proteomics comparison included the highest number of GO terms (59, 20.6%), and the transcriptomics and proteomics comparison included the lowest number, with only 5 (1.7%). The most representative GO terms common to the three omics data were *ribosome*, *structural constituent of ribosome* and *translation*. Between the transcriptomics and epitranscriptomics data, *mRNA binding* and *glutamine metabolic process* were the main GO terms. In the transcriptomics and proteomics comparison, *oxoacid metabolic process* was the main GO term. Finally, between epitranscriptomics and proteomics the main GO terms were *small ribosomal subunit*, *GTPase activity*, *ribosome assembly*, *defense response* and *response to external stimulus*. According to these functional results, several transcripts coding for eukaryotic ribosomal proteins (38) and translation factors (8) had differential epitranscriptomic marks based on the Tombo results (**Figure 5D**).

## Discussion

The response of maritime pine roots to ${\text{NH}}_{4}^{+}$ nutrition has been studied from a multiomics perspective that includes direct RNA sequencing using the ONT platform, which has allowed a global epitranscriptomics analysis. Although N is an essential nutrient for proper plant growth and development (Xu et al., 2012), little is known about the role of epitranscriptomic marks in the regulation of gene expression in response to N nutrition. The results in the present work highlight the importance of epitranscriptomic marks in the regulation of gene expression.

### Epitranscriptome changes in response to NH_4_^+^nutrition

Correlation of global epitranscriptomics results with transcript abundance (**Figure 2C**), especially m^6^A modifications (**Figure 3D**), are consistent with those from previous works in *Populus* (Gao et al., 2021) and *Arabidopsis* (Luo et al., 2014; Wan et al., 2015) and support the role of m^6^A in mRNA turnover, as previously described in mammals (Wang et al., 2014; Li et al., 2019). Additionally, m^6^A identification revealed that in the roots of maritime pine, AAACA and AAACU were the most abundant RRACH 5-mer motifs (**Figure 3C**), as previously reported in *Arabidopsis*, maize, and poplar (Wan et al., 2015; Miao et al., 2020; Parker et al., 2020; Gao et al., 2021). These findings strongly suggest conservation of the RNA m^6^A methylation machinery in plants. However, pine m^6^A distribution, with similar levels of enrichment in the middle of the CDS and at beginning of the 3’-UTR (**Figures 3A, B**), slightly differs from the obtained results in angiosperms, where a greater enrichment of m^6^A was observed in the 3’-UTR (Miao et al., 2020; Parker et al., 2020; Gao et al., 2021). This discrepancy may be attributed to the lack of a well-curated reference genome for maritime pine. Interestingly, the distribution of m^6^A sites in pine transcripts is dynamically regulated by increasing the m^6^A deposition in the 3’-UTR in response to ${\text{NH}}_{4}^{+}$ , which seems to be correlated with poly(A) length and changes in protein abundance (**Figures 5C, D**), as well as the identification of transcripts with both up- and down-regulated epitranscriptomic marks (**Supplementary Dataset 11**). This observation agrees with previous results in other eukaryotic organisms (Meyer et al., 2012; Anderson et al., 2018).

Regarding poly(A) tail length, a previous work in *Caenorhabditis elegans* reported that highly expressed transcripts contained a relatively short and well-defined poly(A) tail (Lima et al., 2017), which has been related to translational efficiency (Wu et al., 2020). In yeast, it was demonstrated that poly(A) tail lengths also have negative correlations with transcript abundance (Tudek et al., 2021). Our results reveal that increased poly(A) tail lengths correlated with higher m^6^A and lower transcript abundances (**Figures 3F, G**). These findings are consistent with the effect of m^6^A modifications on transcript levels (**Figure 3D**), which follow the same trend as previously published results (Luo et al., 2014; Wan et al., 2015; Parker et al., 2020; Gao et al., 2021). The integration of all these results with the positive correlations of m^6^A ratios and poly(A) tail length with protein amounts (**Figures 4C, D**) suggests that m^6^A could affect the translation efficiency of the differentially modified transcripts, as previously described in mammals (Meyer, 2019). This evidence could also explain, at least in part, the lack of a relationship between the transcriptomic and proteomic data.

Epitranscriptomic marks, mainly m^6^A, seem to modulate protein synthesis through mRNA stability and modify translation processes. This is in line with a previous hypothesis considering that initial responses caused by environmental factors are modulated at the level of final products of gene expression to maintain cellular homeostasis (Cañas et al., 2015). Thus, the variable transcriptomic response can be controlled through intermediate steps, such as epitranscriptomic regulation, to generate an appropriate, quick, and stable cellular response.

### Carbon and nitrogen metabolism

**Figure 6:**
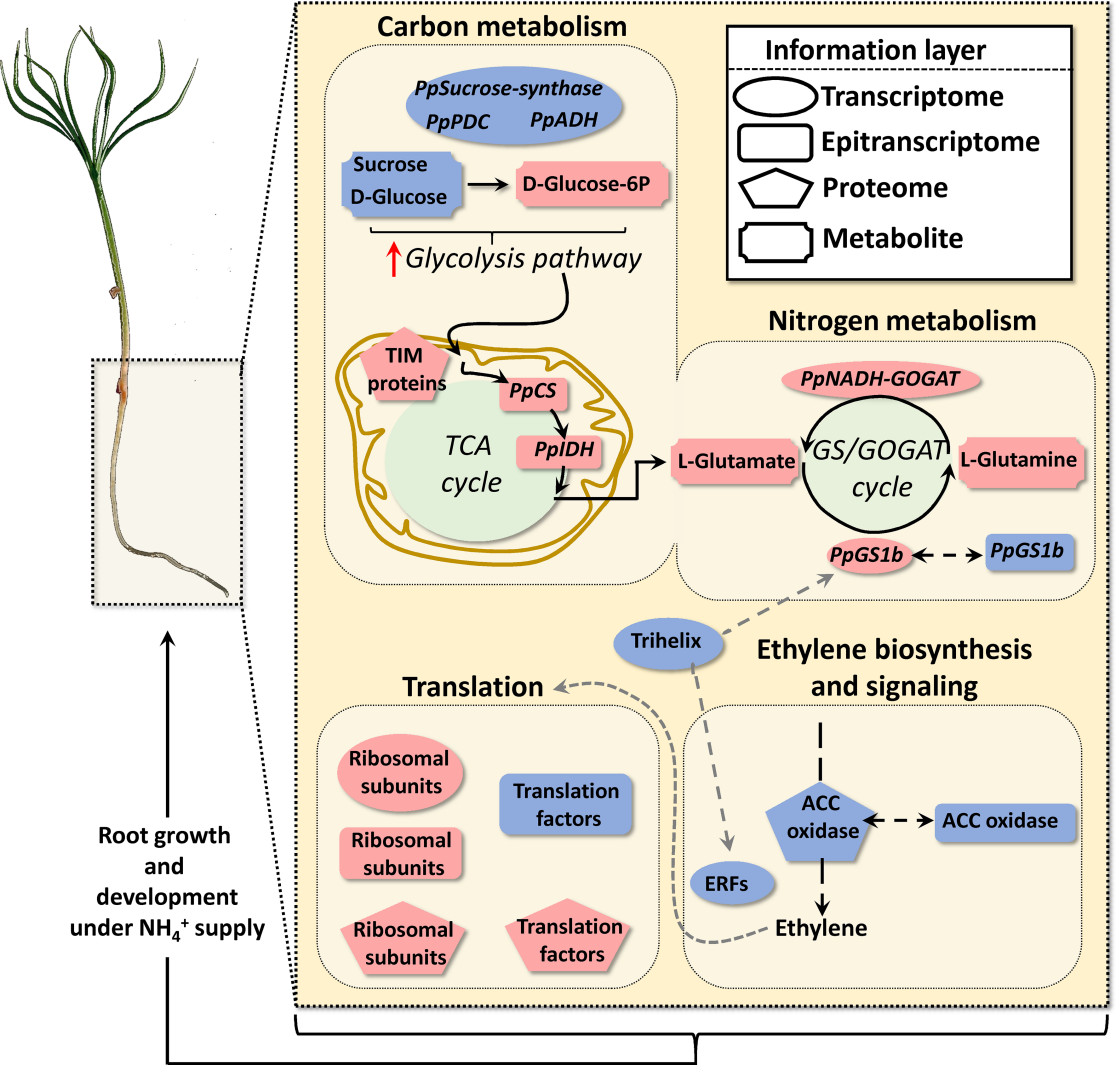
Schematic representation of functional results obtained through the omics integrative approach. ${\text{NH}}_{4}^{+}$ triggers carbon and nitrogen metabolism, ethylene biosynthesis and signaling and translation responses. Geometric red and blue forms indicate upregulation and downregulation respectively of transcripts, RNA nucleoside modifications, proteins, and metabolites.

As expected, N assimilation was induced by ${\text{NH}}_{4}^{+}$ , as shown by the significant increase in *GS1b* and *NADH-GOGAT* transcripts (**Figure 6**; **Supplementary Dataset 3**), consistent with previous transcriptomic reports (Li et al., 2017; Sun et al., 2017). However, no overrepresentation of GS1b and NADH-GOGAT was observed at the proteomic level (**Supplementary Dataset 14**), even though a trend towards higher GS activity was observed in the presence of ${\text{NH}}_{4}^{+}$ (**Supplementary Figure 8**). Similar proteomics results for ${\text{NH}}_{4}^{+}$ nutrition have been described before (Marino et al., 2016; Coleto et al., 2019), as well as the lack of correlation between relative expression levels of *GS* transcripts and GS activity and Gln/Glu levels. To explain these discrepancies, it was hypothesized that the response to N nutrition could be regulated through a posttranscriptional (translational or posttranslational) mechanism (Ortigosa et al., 2020). In this way, several nucleosides with differential epitranscriptomic marks, including m^6^A, have been identified in *GS1b* transcripts, which could explain the differences between transcriptomics and proteomics data (**Supplementary Datasets 10 and 13**). Accordingly, in *Arabidopsis*, m^6^A can regulate the alternative polyadenylation and transcript abundance of the *GLN1;1* and *GLN1;3* genes during nitrate assimilation and signaling (Hou et al., 2021).

Additionally, the results of this study suggest the existence of a carbon flux through glycolysis and the TCA cycle to provide carbon skeletons for N assimilation (de la Peña et al., 2019). Interestingly, the fermentation pathway seems to be repressed, as suggested by the downregulation of pyruvate decarboxylase (PDC) transcripts and the decrease in alcohol dehydrogenase (ADH) and aldehyde dehydrogenase (ALDH) protein levels (**Supplementary Datasets 2 and 14**). It is well known that plants under low oxygen conditions regulate their metabolism, inducing the fermentation pathway in which PDC and ADH activities are essential, which results in pyruvate consumption, the production of ethanol and the concomitant oxidation of NADH to NAD^+^ (Mithran et al., 2014). The consumption of soluble sugars (sucrose and D-glucose) (**Supplementary Figure 1** and **Supplementary Dataset 1**), the repression of the fermentative pathway and the overrepresentation of TIM proteins (inner membrane translocase proteins) (**Supplementary Dataset 14**) highlight the need for organic acids from TCA cycle to assimilate ${\text{NH}}_{4}^{+}$ and produce energy for plant growth. In addition, our results suggest that the biosynthesis of organic acids could be regulated through the epitranscriptome according to the differentially overmodified sequence found in the coding transcript for the pyruvate kinase enzyme (pp_236058), which could imply an additional regulation mechanism together with those previously described (Wulfert et al., 2020).

### Ethylene

Transcriptomic studies carried out in rice described that under excess ${\text{NH}}_{4}^{+}$ , ethylene (ET) could be one of the major regulatory molecules in roots (Sun et al., 2017). Additionally, Li et al. (2013) described in *Arabidopsis* that shoot-supplied ${\text{NH}}_{4}^{+}$ promoted ET biosynthesis only in shoots, resulting in a reduction in the lateral root formation process due to *auxin transporter 1* (*AUX1*) repression. These data might indicate that ET biosynthesis is involved in detrimental ${\text{NH}}_{4}^{+}$ -related phenotypes, such as a reduction in the number of lateral roots (LRs). Some reports have described that the application of inhibitors of the ET biosynthesis relieved symptoms of ${\text{NH}}_{4}^{+}$ toxicity affecting RSA (Li et al., 2013). However, previous results in maritime pine seedlings indicated that ${\text{NH}}_{4}^{+}$ promotes root growth (Ortigosa et al., 2020) and a wide repression of the ACC oxidase in the root apex during early transcriptome response (Ortigosa et al., 2022).

A comparison of the root transcriptomic response and differential proteomics results revealed that ${\text{NH}}_{4}^{+}$ promotes a decrease in ET-related transcripts and proteins. Several ET-responsive TFs were downregulated at the transcriptomic level, while proteomic results revealed that ACC oxidase was downregulated (**Supplementary Datasets 2** and **14**). ACC oxidase is the enzyme responsible for the final step in the biosynthesis of ET in plants (John et al., 1999). Furthermore, ACC oxidase transcripts exhibited a nucleoside putative modification in the 3’-UTR (position 462) that was differentially increased under ${\text{NH}}_{4}^{+}$ treatment (**Supplementary Dataset 16**). Since the levels of ACC oxidase transcripts had no significant changes, it is possible that this epitranscriptomic mark could be involved in the translational regulation of these transcripts, therefore affecting the ET levels of the organ. Interestingly, it is reasonable to think that this kind of response could be related to conifer preference for ${\text{NH}}_{4}^{+}$ . Additional technical approaches are required to identify the exact modification and validate its biological relevance in the regulation of ACC oxidase and ET biosynthesis. Finally, the relationship between ET and N nutrition is remarked by the differential expression (negative) of a trihelix family TF that has a very high similarity degree to *AT3G54390* in *Arabidopsis* (**Figure 6**). This TF in *Arabidopsis* has been shown to interact with the promoters of genes involved in N metabolism and ET biosynthesis including *glutamine synthetase* (AT1G66200), *nitrite reductase* (AT2G15620), *ET-responsive transcription factor* (*ERF012*; AT1G21910) and *1-aminocyclopropane-1-carboxylate synthase* (AT5G65800) (Gaudinier et al., 2018).

### Translation and growth

The results obtained also indicate a reconfiguration of the ribosomal proteins and elongation factors at all biological levels, although this was more visible in epitranscriptomics and proteomics results (**Figures 5D**, **6**; **Supplementary Datasets 2, 10, 14** and **17**). This process is clearly related to the correlation between m^6^A ratios and protein amounts (**Figure 4C**), suggesting that ${\text{NH}}_{4}^{+}$ nutrition promotes general translation activation to support the root growth of maritime pine seedlings. This kind of effect on the ribosomal protein composition and proteins involved in translation has been previously observed under different conditions, including plant mineral nutrition (Wang et al., 2013; Prinsi and Espen, 2018; Ferretti and Karbstein, 2019). The data suggest that changes in the profiles of ribosomal proteins can be significantly mediated by epitranscriptomic modifications of their transcripts. These posttranscriptional changes can influence ribosome function and performance. One example is the RACK1 proteins, which are components of the small subunit of the ribosome and are involved in translation quality control promoting the endonucleolytic cleavage of nonstop mRNA (Ikeuchi and Inada, 2016). These proteins in plants are involved in the response to phytohormones, including ET, and in the regulation of growth and development (Wang et al., 2019). Interestingly, the mRNA of a *RACK1* gene in maritime pine roots has several differential epitranscriptomic marks (**Supplementary Datasets 10** and **11**). Although it was not significant, RACK1 protein expression was repressed under ${\text{NH}}_{4}^{+}$ conditions, suggesting a change in the quality control mechanisms and even in the integration of the plant signaling pathways. This result agrees with the negative regulation of ACC oxidase and repression of several ERFs (**Supplementary Datasets 2** and **14**), since ET signaling affects gene translation in different ways (Merchante et al., 2015).

Additionally, related to the regulation of translation and root growth, among the transcriptomic results, the repression of an *FCS-like zinc finger* was observed (**Supplementary Dataset 2**). FCS proteins interact with SnRK1 to mediate the interaction of SnRK1 with other proteins (Jamsheer et al., 2018). SnRK1 is a kinase that phosphorylates RAPTOR, a regulatory element of the TOR complex, repressing TOR activity (Fu et al., 2020). Considering the ribosomal protein changes and the increase in root growth observed under ${\text{NH}}_{4}^{+}$ nutrition in maritime pine seedlings (Ortigosa et al., 2020), it can be hypothesized a decrease of SnRK1 activity over the TOR complex in these conditions. The TOR complex integrates different developmental and environmental signals, including nutritional status, promoting ribosome biogenesis, translation, and plant growth (Henriques et al., 2022). Finally, a transcript coding for a TCTP protein had several differential modifications (**Supplementary Dataset 11**). It is well established in plants that *TCTP* mRNA is synthetized in shoots and transported to roots, where it is translated and promotes lateral root formation (Branco and Masle, 2019). Interestingly, transport of this mRNA is mediated through epitranscriptomic marks (Yang et al., 2019). This suggests additional mechanisms controlling growth and development in response to N nutrition in maritime pine roots.

Therefore, the results presented in this work suggest that protein translation and growth are finely regulated through epitranscriptomic marks, including m^6^A, to acquire an optimum response to N supply (**Figure 6**). More research efforts are required to corroborate this hypothesis and to investigate whether ET could act as a modulator of the integrated response observed due to its effects on the translation process.

## Conclusions

Finally, and according to our results in maritime pine, ${\text{NH}}_{4}^{+}$ triggers a root systemic response at short-term that mainly involved changes in key pathways such as carbon and nitrogen metabolism, ET signaling pathway, translation and root growth (**Figure 6**). Interestingly, ET-related response observed was different from that of previously reported in other ${\text{NH}}_{4}^{+}$ tolerant plants such as rice (Sun et al., 2017) and supports previous findings in maritime pine (Ortigosa et al., 2022). Additionally, the obtained results strongly suggest that protein translation is finely regulated through the epitranscriptome affecting mRNA turnover and probably the ribosome performance. Although the gene transcription is very reactive to development and environmental changes the processes that regulate mRNA metabolism and translation, such as epitranscriptomics marks, can modulate the response buffering, filtering, and focusing the final products of the gene expression. In this case, the epitranscriptomic regulation seems directed to acquire a proper response to promote root growth under ${\text{NH}}_{4}^{+}$ supplementation.

## Data availability statement

The datasets presented in this study can be found in online repositories. The names of the repository/repositories and accession number(s) can be found in the article/**Supplementary Material**.

## Author contributions

FO have performed the experiments; CL-F has performed the bioinformatic analyses; JP-C and FRC have performed the statistical data analysis; FO and RC have wrote the manuscript; CA and FMC made additional contributions and edited the manuscript. FO and RC have planned and designed the research. RC, CA, and FMC were the responsible of the funding acquisition. All authors contributed to the article and approved the submitted version.
