# Supplementary material for: Epitranscriptome changes triggered by ammonium nutrition regulate the proteome response of maritime pine roots
Source: Front Plant Sci. 2022 Dec 22;13:1102044. doi: 10.3389/fpls.2022.1102044 (PMC9815506; doi:10.3389/fpls.2022.1102044)
Supplement: Supplementary file 4 [file DataSheet_4.zip › Supplementary_Figures.pdf]

Supplementary Material

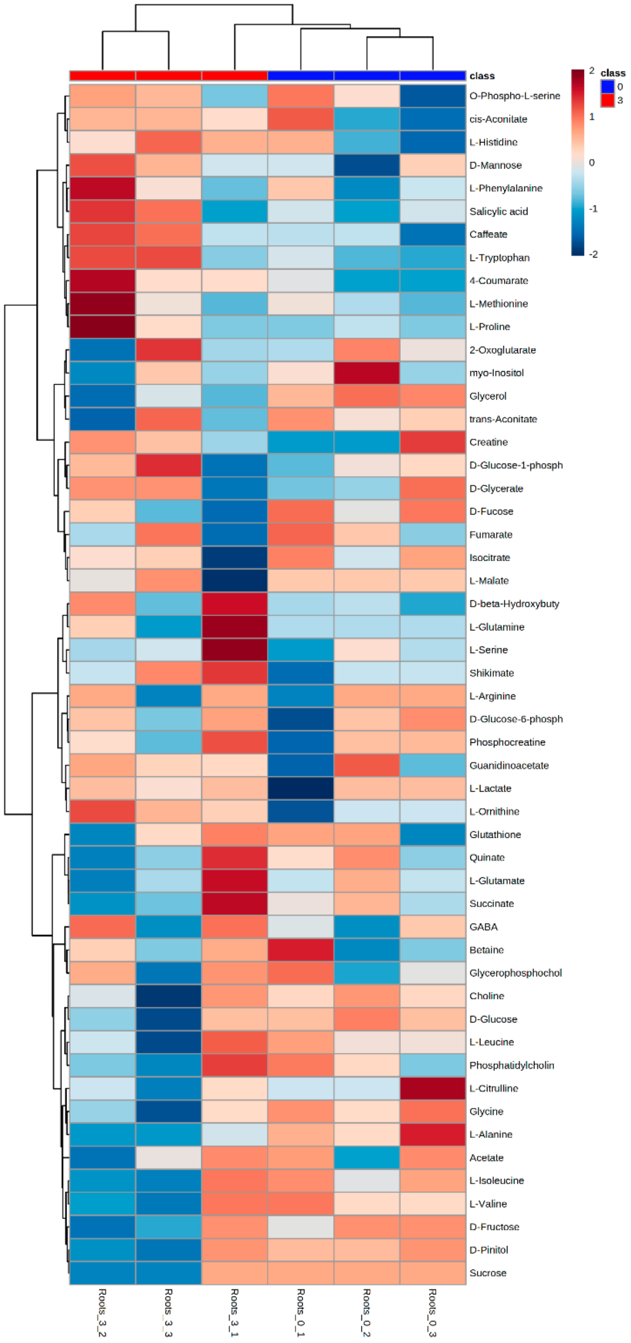

Supplementary Figure 1. Heatmap of root metabolites. The analysis was done with Metaboanalyst using the Euclidean distance and Ward clustering algorithm. Class 0 (blue group) corresponds with control samples irrigated with distilled water. Class 3 (red group) corresponds with seedlings irrigated with 3 mM NH_4^+.

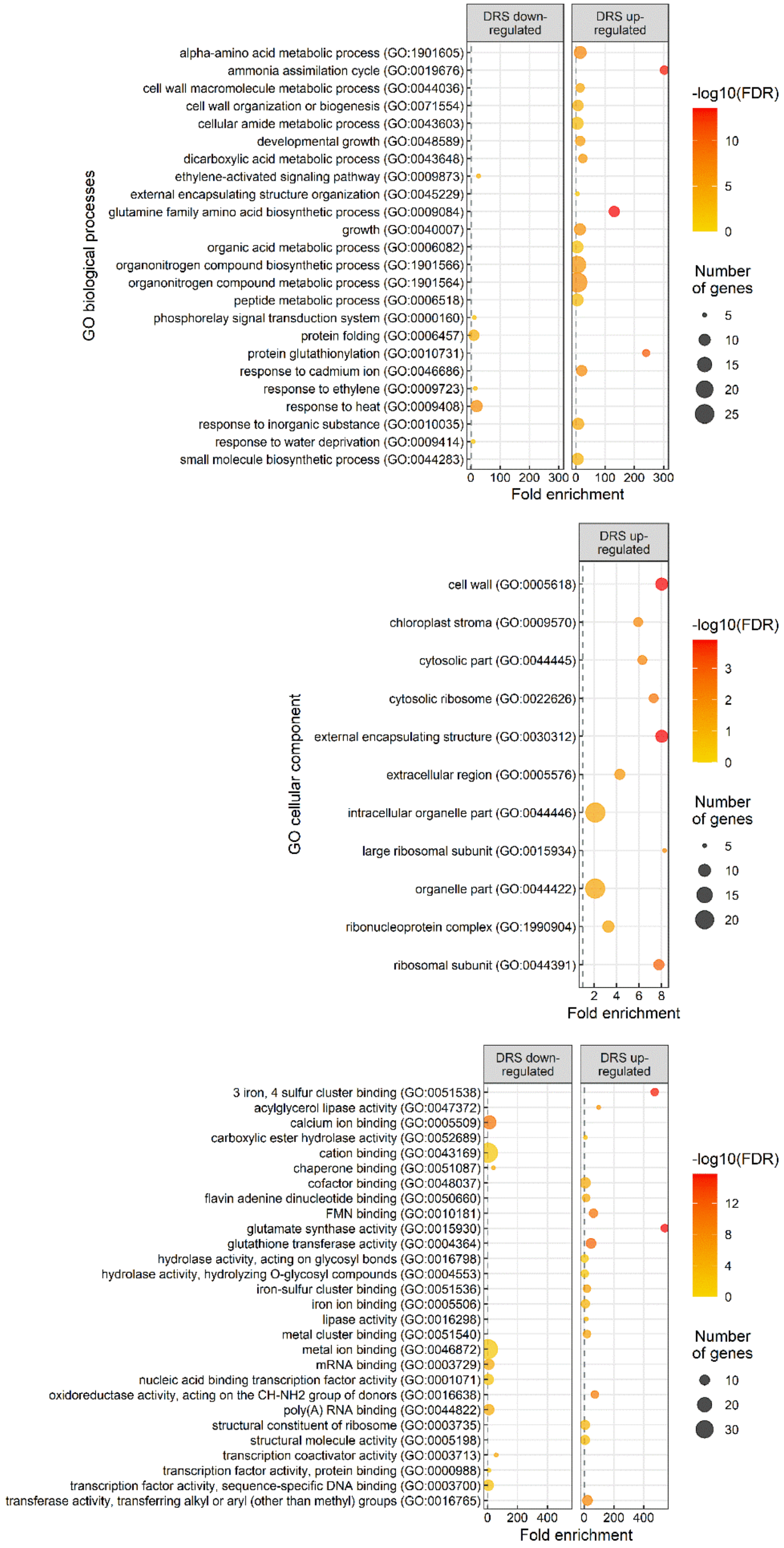

Supplementary Figure 2. Significant GO terms after SEA analysis using the DRS results.

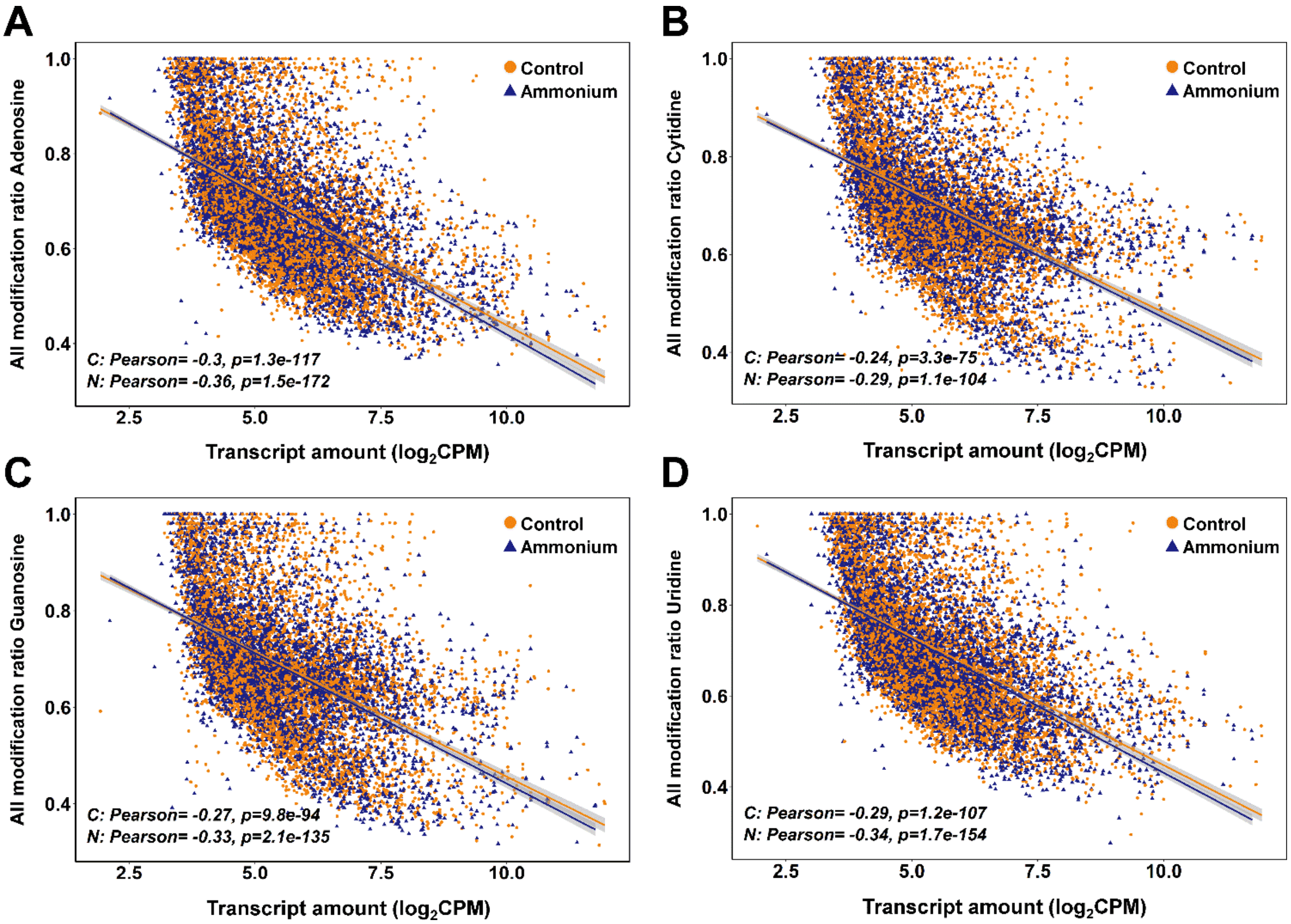

Supplementary Figure 3. Scatter plot and correlations between the transcript amounts and the modification ratios detected using Tombo software for each nucleoside. Epitranscriptomic modifications in adenosines (A), cytidines (B), guanosines (C) and uridines (D).

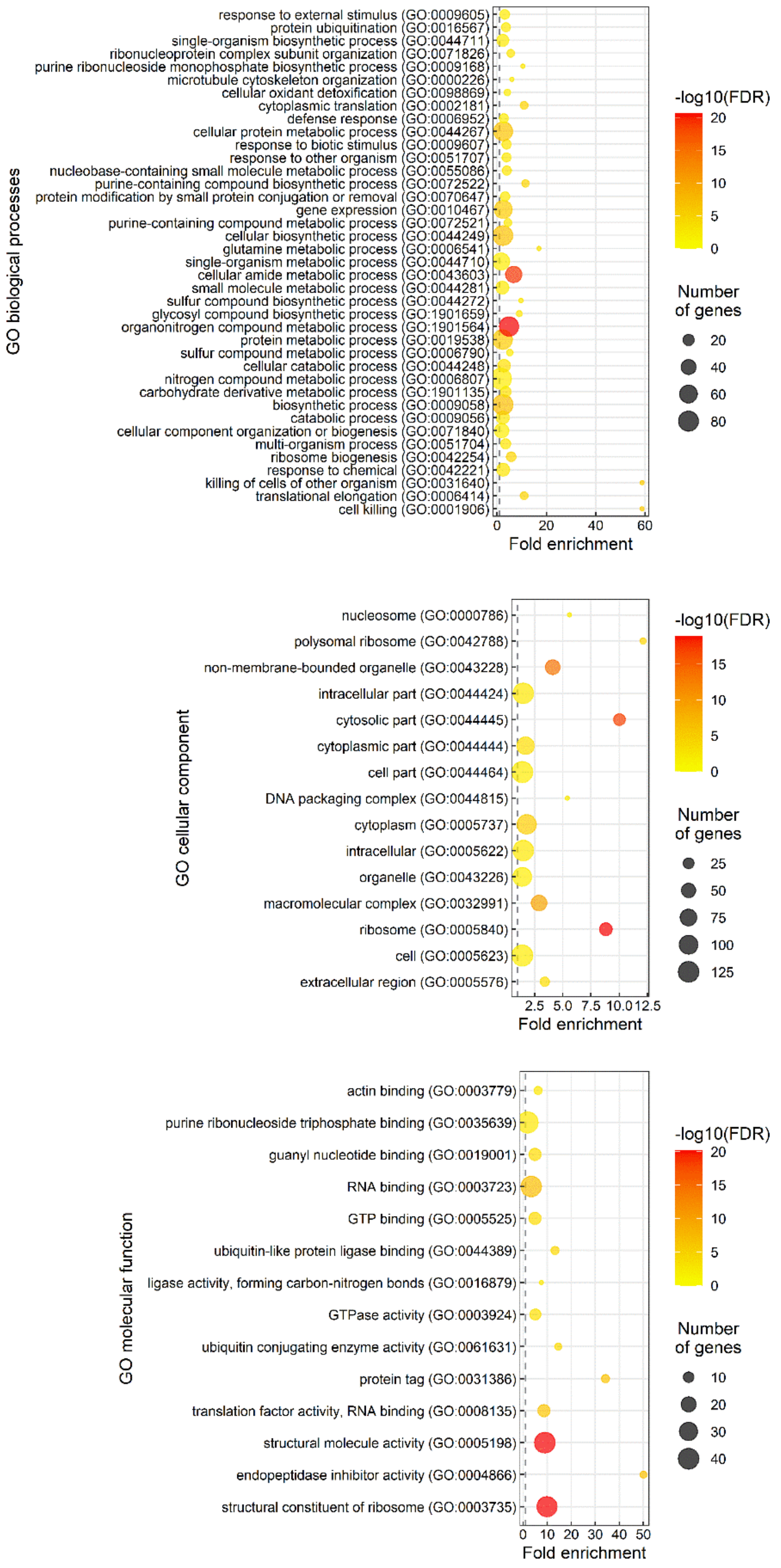

Supplementary Figure 4. Significant GO terms after SEA analysis using the epitranscriptomics results obtained from Tombo software.

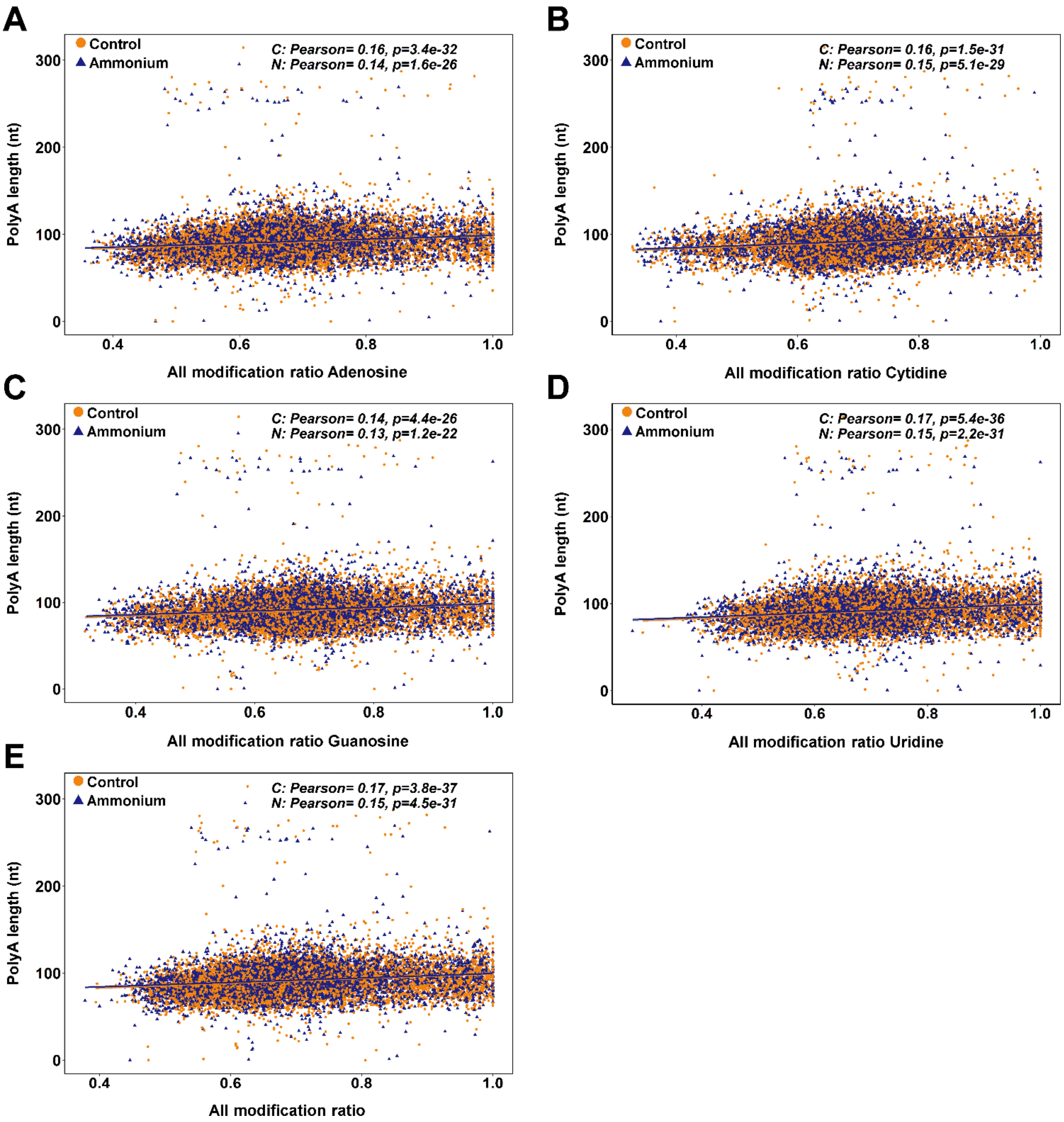

Supplementary Figure 5. Scatter plot and correlations between the poly(A) length and the modification ratios detected using Tombo software for each nucleoside and global results. Epitranscriptomic modifications in adenosines (A), cytidines (B), guanosines (C) uridines (D) and all nucleosides (E).

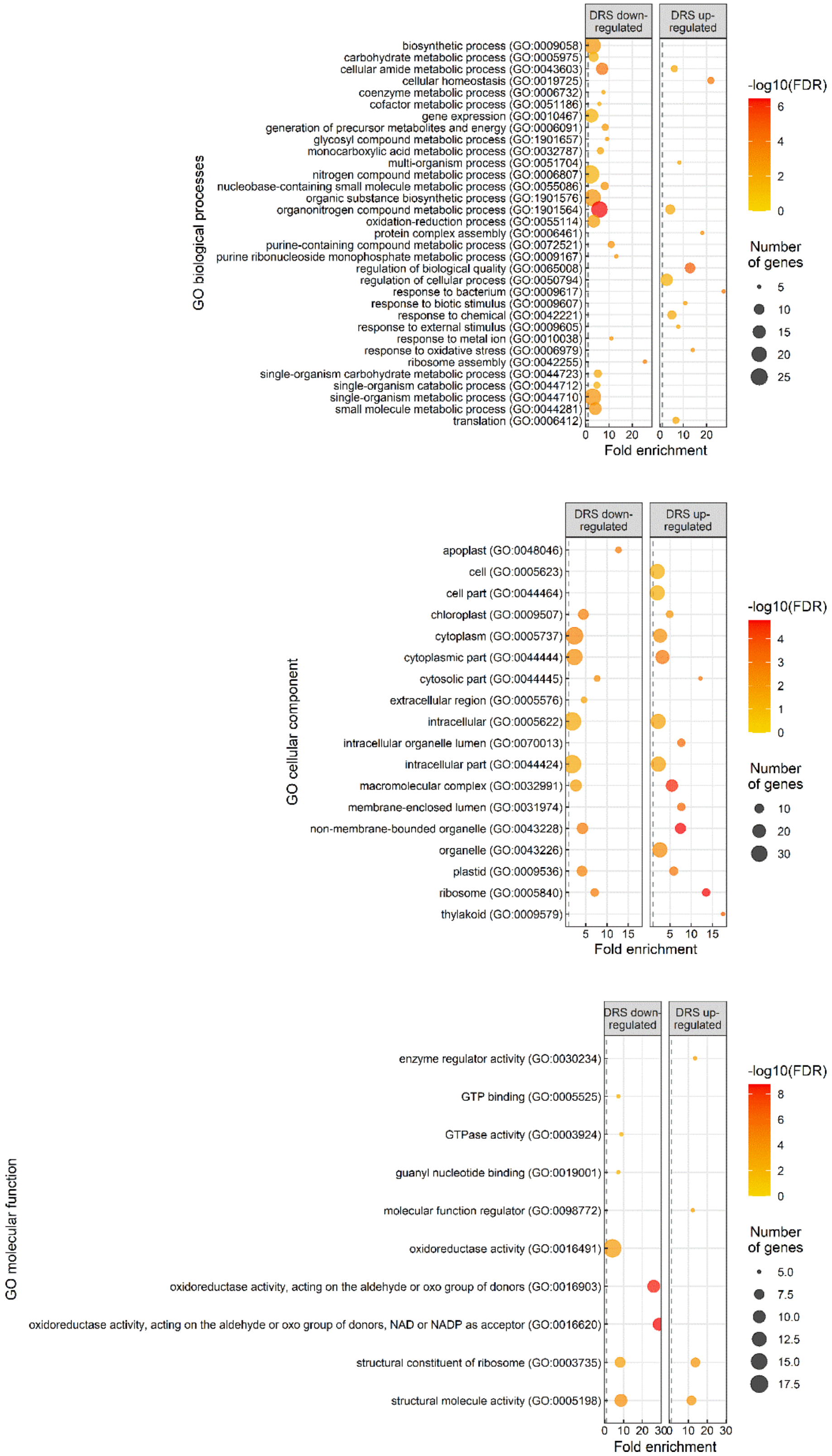

Supplementary Figure 6. Significant GO terms after SEA analysis using the proteomics results.

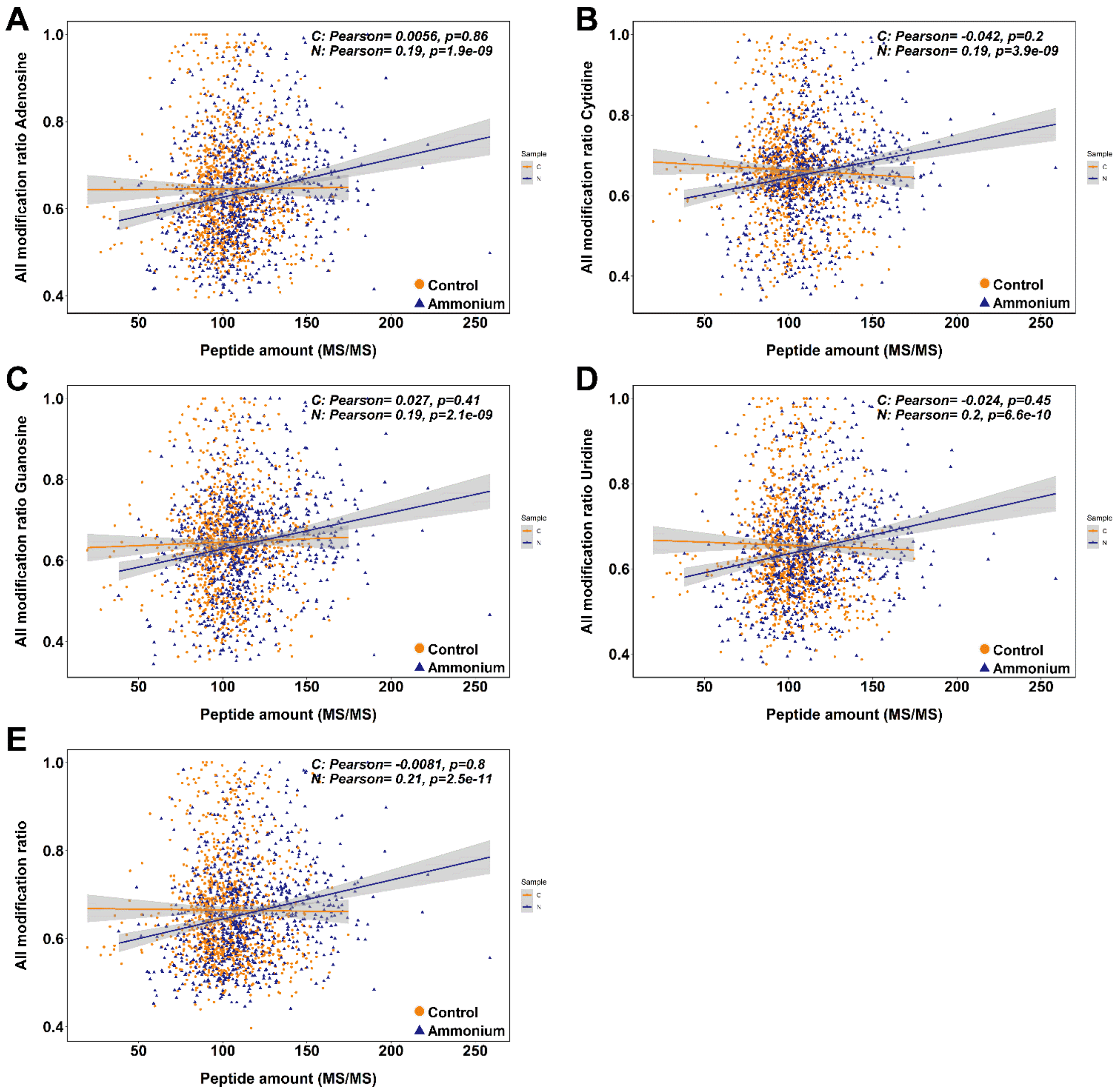

Supplementary Figure 7. Scatter plot and correlations between the protein amounts and the modification ratios detected using Tombo software for each nucleoside and global results. Epitranscriptomic modifications in adenosines (A), cytidines (B), guanosines (C) uridines (D) and all nucleosides (E).

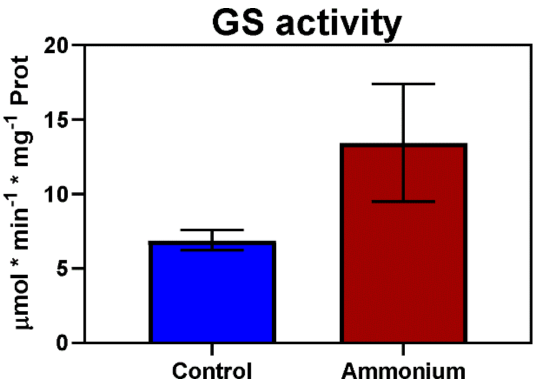

Supplementary Figure 8. Glutamine synthetase activity in maritime pine roots under ammonium nutrition. The results are the mean of three independent experiments using biological pools. Error bars correspond to SE. Enzymatic determinations were described in Supplementary Methods 1.
